# CO_2_ reforming of benzene into syngas by plasma-enhanced packed-bed dielectric barrier discharge with different packing materials

**DOI:** 10.3389/fchem.2025.1532478

**Published:** 2025-03-05

**Authors:** Yafeng Guo, Shiye Cheng, Yu Du, Na Lu, Chao Li, Hanchun Bao, Xiao Zhu, Shi-Ya Tang

**Affiliations:** ^1^ State Key Laboratory of Chemical Safety, Qingdao, China; ^2^ SINOPEC Research Institute of Safety Engineering Co., Ltd, Qingdao, China; ^3^ School of Electrical Engineering, Dalian University of Technology, Dalian, China

**Keywords:** non-thermal plasma (NTP), CO_2_ reforming, syngas, tar reforming, DBD (dielectric barrier discharge) reactor

## Abstract

Tar reforming has gained widely attention in the field of biomass gasification. Dielectric barrier discharge (DBD) presents a promising technology for the conversion of biomass gasification tar under ambient conditions. In this study, plasma-enhanced dual DBD (ED-DBD) combined with packing materials such as glass (SiO_2_) beads and SiC blocks was utilized to examine the CO_2_ reforming of benzene, serving as a tar analogue, into syngas. (Introduction) First, the discharge characteristics and performance metrics for benzene and CO_2_ conversion (Method 1) were evaluated and compared between the conventional dual dielectric barrier discharge (D-DBD) system and the ED-DBD reactor, which was augmented with SiO_2_ beads and SiC blocks. The findings indicated that the ED-DBD reactor incorporating SiC blocks demonstrated superior performance, achieving a benzene conversion of 51.0%, a CO_2_ conversion of 75.0%, and an energy efficiency for CO_2_ conversion of 73.9%. The results satisfy the minimum requirements for CO_2_ conversion and energy efficiency required for industrial application (Results and Discussion 1). Secondly, analysis via X-ray Photoelectron Spectroscopy (XPS) (Method 2) revealed that a minor proportion of carbon elements originating from the SiC blocks within the plasma region were involved in the reaction process (Results and Discussion 2). Moreover, an elevated initial concentration of CO_2_ in the benzene system enhanced the degradation of benzene, whereas the introduction of benzene into the CO_2_ system promoted the conversion of CO_2_. Emission spectroscopy (Method 3) corroborated the presence of active hydroxyl radical (·OH) particle during the discharge process. It suggests that the SiC-packed ED-DBD reactor more efficiently generates active OH particles during the discharge compared to the SiO_2_-packed ED-DBD reactor (Results and Discussion 3). This study not only offers an effective method for converting tar analogues into syngas under mild conditions but also presents an alternative approach for CO_2_ utilization within a carbon-neutral strategy.

## 1 Introduction

Biomass is extensively acknowledged as a fundamental source of renewable energy, originating from plant materials and forestry residues. Biomass gasification technology provides an efficient method for converting biomass into syngas (comprising hydrogen and carbon monoxide) or other gaseous fuels. Nevertheless, the gasification process unavoidably generates tar as a by-product, which poses significant risks of equipment corrosion and pipeline obstructions. Thus, the elimination of biomass tar has emerged as a critical challenge in the progression of biomass gasification technologies. Conventional methods for tar removal encompass both physical and chemical strategies. Physical techniques like extraction may result in secondary pollution, whereas pyrolysis demands temperatures of no less than 850°C (Phuphuakrat et al., 2010), and catalytic cracking requires a minimum temperature of 450°C to effectively activate the catalyst. (Bosmans et al., 2013; Ren et al., 2022).

Non-thermal plasma technology has recently gained significant attention in the fields of material modification, agriculture, and medicine (Bekeschus et al., 2020; Chen et al., 2018; Yu et al., 2020), owing to its high chemical activity. Numerous efforts have been devoted to tar removal using plasma. Nair et al. have investigated the decomposition of various tar analog compounds (e.g., naphthalene, toluene, phenol) using pulsed corona plasma and found that naphthalene exhibited a higher reaction rate of decomposition compared to phenol (Nair et al., 2003; Nair et al., 2005). Tu et al. have utilized hybrid gliding arc plasma to reform toluene and naphthalene as tar model compounds, achieving a toluene conversion of 95.7% and a naphthalene conversion of 83.4% (Ashok and Kawi, 2013; Mei et al., 2019a; Mei et al., 2019b). In addition to corona and gliding arc plasmas, dielectric barrier discharge (DBD) has emerged as a commonly used atmospheric plasma at room temperature, known for its user-friendly operation. Kim et al. have compared the performance of different DBD reactors for tar decomposition and found that blank DBD demonstrated the best performance, achieving nearly 75% degradation of benzene in N_2_/O_2_ carrier gas, which is the major organic component (37.9%) of the biomass gasification tar (Saleem et al., 2020). The degradation of benzene requires a higher specific energy density of the discharge. The dielectric nature of the packing materials, such as beads or pellets, in the dielectric barrier discharge (DBD) reactor leads to polarization when subjected to the electric field applied between the electrodes. Consequently, opposite charges accumulate at the contact interfaces between the beads, which may result in a significant local enhancement of the electric field within the plasma (Bogaerts et al., 2019). The influence of the packing material in the DBD discharge area on the conversion of benzene remains inadequately understood.

Moreover, the oxidation reaction of benzene to syngas (H_2_ and CO) using CO_2_, one of the products of biomass gasification, presents a potential method for converting intermittent sustainable electricity into storable chemical energy. Xiao et al. demonstrated that the presence of CO_2_ greatly promoted toluene degradation, and the combination of DBD and manganese-based catalyst increased the maximum CO yield from 16.5% to 28.8% (Xiao et al., 2021). Yin et al. suggested that achieving a 60% conversion of CO_2_ along with high energy efficiency should be an aspirational target for future research, although few previous studies have managed to surpass both of these thresholds (Olivier et al., 2023).

In order to examine the impact of the packing materials on the CO_2_ reforming of benzene, a plasma-enhanced dual DBD (ED-DBD) incorporating two distinct packing materials, namely, SiO_2_ beads and SiC blocks, was utilized. Initially, the discharge voltage and current waveforms of the ED-DBD, in conjunction with the selected packing materials, were analyzed and compared to those of the conventional dual dielectric barrier discharge (D-DBD). Subsequently, the performances of benzene degradation and CO_2_ conversion were assessed using SiO_2_ beads and SiC blocks. Additionally, optical emission spectroscopy was employed to further investigate the system.

## 2 Methods

### 2.1 Experimental set-up

**FIGURE 1:**
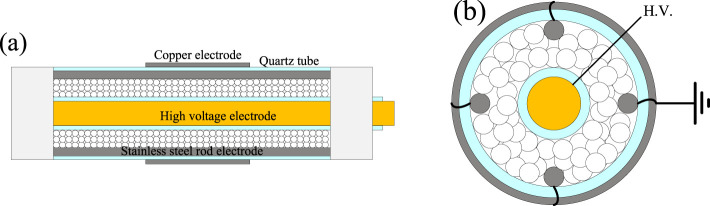
Schematic view of ED-DBD reactor (side view **(a)** and top view **(b)**).

The experiments were conducted in an ED-DBD reactor, consisting of two coaxial quartz tubes with a wall thickness of 1 mm and three electrodes, as shown schematically in Figure 1. The reactor was filled with SiO_2_ beads or SiC blocks, and the discharge gap between the tubes was 3.5 mm. The inner tube was filled with a copper rod with a diameter of 5 mm as a high-voltage electrode. The ED-DBD reactor was tightly wrapped around a copper sheet with a length of 6 cm, as the first ground electrode. Four stainless steel electrodes (2 mm in diameter) were added to the discharge gap as the second ground electrodes, which were closely fitted to the inner wall of the outer quartz tube and paralleled to the high-voltage electrode. The packing material (i.e., SiO_2_ beads and SiC blocks) was used in the size fraction of 1–2 mm filled the entire discharge gap.

**FIGURE 2:**
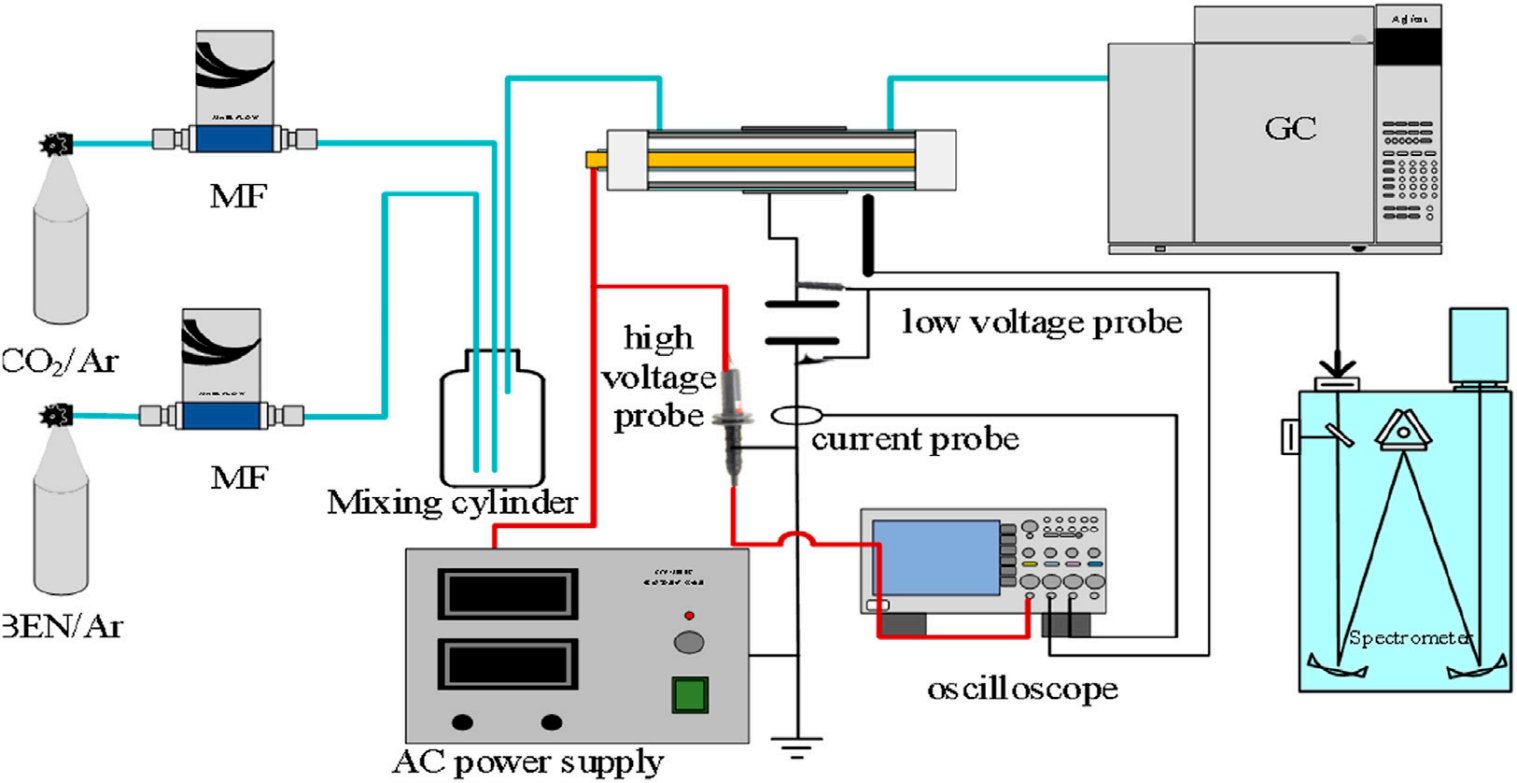
Schematic view of experimental set-up.

The experimental system, as shown in Figure 2, consisted of the gas supply, discharge and detection systems.

The gas supply system used high-purity argon (99.999%, 13.5 MPa, Shandong Honda Biotechnology Co., Ltd.), benzene (0.06%, the rest was argon, 5.0 MPa, Shandong Hongda Biotechnology Co., Ltd.), and CO_2_ (1.0%, the rest was argon, 10.0 MPa, Shandong Hongda Biotechnology Co., Ltd.) as background gas, model molecule, and oxidizing agent, respectively. CO_2_ and benzene were mixed in a gas mixing bottle to obtain an initial concentration of 3,000 ppm CO_2_ and 300 ppm benzene. The total gas flow rate was 250 mL/min.

The discharge system utilized high-voltage AC power supply (CTP-2000K, Nanjing Suman Plasma Technology Co., Ltd.) to drive the electric field local enhanced DBD reactor, with a discharge voltage range of 4–12 kV and discharge frequency range of 6–10 kHz. The discharge voltage and current waveforms were detected using a high-voltage probe (Tektronix, P6015A) and current probe (Tektronix, TCP0030A), and the oscilloscope (Tektronix, MDO3054) recorded the voltage, frequency, current, and other discharge data of the reactor discharge process.

The product detection system consisted of a gas chromatograph (Agilent GC 7890B with FID and TCD detectors) and an emission spectrometer (Princeton Instruments, SP-2750). The benzene concentration was quantitatively detected using a DB-HeavyWax (J&W 123–7163) column with a high-purity nitrogen carrier gas (99.999%, 13.5 MPa, Shandong Hongda Biotechnology Co., Ltd.). The vaporization chamber temperature was set at 250°C, the column box temperature was initially set at 40°C for 5 min, then raised to 100°C at a rate of 15°C/min for 2 min, and the FID detector operating temperature was 300°C with hydrogen flow rate of 32 mL/min (99.999%, 13.5 MPa, Shandong Hongda Biotechnology Co., Ltd.) and an air flow rate of 400 mL/min (99.999%, 13.5 MPa, Shandong Hongda Biotechnology Co., Ltd.). The CO_2_ concentration was detected using a GS-CarbonPLOT (Agilent 113–3133) column with a vaporization chamber temperature of 180°C, a 30:1 shunt, a column box temperature initially set at 40°C for 10 min, and TCD detector temperature of 200°C. The CO and H_2_ concentrations were detected using HP-PlotQ (Agilent 19095P-Q04) and HP-Plot Molesieve (Agilent19091P-MS4) columns in series by chromatographic center cutting technology. The carrier gas was high-purity argon (the specifications were the same as above), with a flow rate of 10.917 mL/min, a vaporization chamber temperature of 180°C, and a column box temperature initially set at 35 °C for 11.5 min. The programmed temperature was then increased to 120°C at a rate of 15°C/min for 10 min, and the TCD detector temperature was 250°C. The tail blowing gas was high-purity argon (the specifications were the same as above), with a reference flow rate of 10 mL/min. The emission spectrum generated an emission spectrum for diagnosing the plasma reaction process, and the characteristic peak wavelength data was used to analyze the possible species in the plasma. The signal in the range of 200–800 nm was collected and processed, with working parameters including an exposure time of 80 m, a light intake slit of 500 μm, and a grating scale of 600 g/mm.

Before each discharge, the reactor was injected with the necessary gas source for 90 min. An infrared camera (FLIR T660) was used to measure the temperature variation of the reactor during the 20-min continuous operation. The temperature of the quartz glass tube on the right side of the first grounding electrode was specifically selected as the focal point, which is not representative for the plasma (gas) itself.

### 2.2 Data analysis

The discharge power was calculated using Equation 1, where *P* (kW) represents the plasma discharge power, *C*_M_ denotes the measurement capacitance, *U* represents the voltage measured by the high voltage probe, *U*_M_ represents the voltage of the measurement capacitance and *f* represents the discharge frequency.

$$P=\frac{1}{T}\int_{0}^{T}UIdt=f\int_{0}^{T}U\;\times \;C_{M}\;\times \;\frac{{dU}_{M}}{dt}dt=fC_{M}\int_{0}^{T}UdU_{M}$$ (1)

The benzene and CO_2_ conversion rate $\Upsilon_{B}$ and $\Upsilon_{{CO}_{2}}$ were calculated using Equations 2, 3, respectively.

$$\gamma_{B}=\frac{{\varphi \left(C_{6}H_{6}\right)}_{\text{in}-}{\varphi \left(C_{6}H_{6}\right)}_{\text{out}\;}}{{\varphi \left(C_{6}H_{6}\right)}_{\text{in}}}\times 100\%$$ (2)

$$\gamma_{{CO}_{2}}=\frac{{\varphi \left(CO_{2}\right)}_{\text{in}}-{\varphi \left(CO_{2}\right)}_{\text{out}}}{{\varphi \left(CO_{2}\right)}_{\text{in}}}\times 100\%$$ (3)

The molar yield of CO and H_2_ were calculated using Equations 4, 5, where $Y_{B}$ represents the molar yield of benzene degradation and $Y_{CO}$ represents the molar yield of CO_2_ conversion.

$$Y_{H_{2}}=\frac{\;{\varphi \left(H_{2}\right)}_{\text{out}}}{3\times {\varphi \left(C_{6}H_{6}\right)}_{\text{in}}}\times 100\%$$ (4)

$$Y_{CO}=\frac{\;{\varphi \left(\text{CO}\right)}_{\text{out}}}{{\varphi \left({\text{CO}}_{2}\right)}_{\text{in}}+6\times {\varphi \left(C_{6}H_{6}\right)}_{\text{in}}}\times 100\%$$ (5)

The energy efficiency of CO_2_ reforming was the ratio of the chemical energy of CO_2_ reforming of benzene harvested to the electrical energy spent,

$$\eta_{CO_{2}}=\frac{\;V\left({2\Delta H_{CO}\cdot Y}_{CO}+0.5{\Delta H_{H_{2}}\cdot Y}_{H_{2}}\right)\times \left({{\varphi \left({\text{CO}}_{2}\right)}_{\text{in}}\varphi \left({\text{CO}}_{2}\right)}_{\text{in}}+{\varphi \left(C_{6}H_{6}\right)}_{\text{in}}\right)}{22.4\times 60\times P\times \left({\varphi \left({\text{CO}}_{2}\right)}_{\text{in}}\right)\times \gamma_{CO_{2}}}\times 100\%$$

In the above equation, *V* (L min^-1^) represents the volumetric flow rate, *P* (kW) represents the plasma discharge power, Δ*H*_CO_ represents the fuel value of CO (283 kJ mol^−1^), Δ*H*_H2_ represents the fuel value of H_2_ (285.8 kJ mol^−1^), and *Y*_CO_ or *Y*_H2_(mol min^−1^) represents the CO or H_2_ yield, $\Upsilon_{CO_{2}}or\;\Upsilon_{B}$ represents CO_2_ or benzene conversion rate. The factor of 22.4 (L mol^−1^) represents gas molar volume constant, and 60 is used to convert seconds into minutes.

## 3 Results and discussion

### 3.1 Input power

**FIGURE 3:**
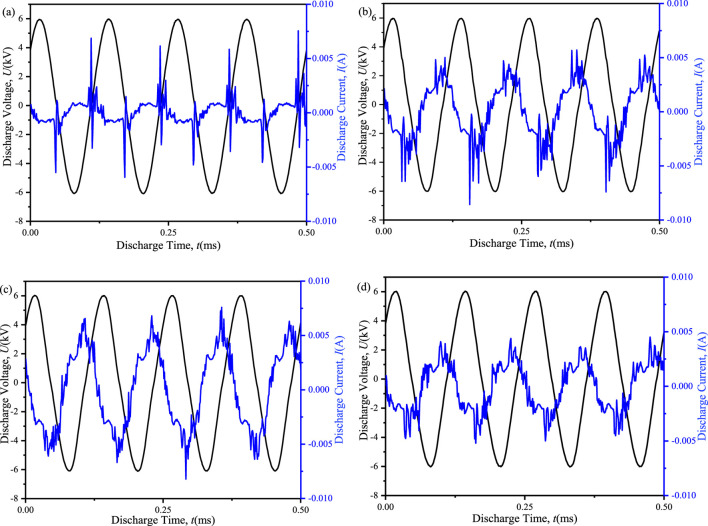
Discharge voltages and currents of different reactors at an applied voltage of 12 kV and a frequency of 8 kHz. **(a)** D-DBD reactor, **(b)** ED-DBD reactor, **(c)** ED-DBD reactor (filled with SiO_2_ beads), **(d)** ED-DBD reactor (filled with SiC blocks).

The input power in dielectric barrier discharges (DBDs) plays a crucial role in determining the number of electrons generated, which subsequently affects the ensuing chemical processes. Figure 3 presented the discharge voltage and current waveforms for both the ED-DBD and D-DBD reactors, as well as the cases with packing materials. Comparing the blank D-DBD and ED-DBD reactors, as illustrated in Figures 3A, B, it was evident that the current pulse of the ED-DBD reactor was six times higher than that of the blank D-DBD reactor. However, this pulse occurred only after the current peak in the D-DBD reactor. Furthermore, the discharge intensity of the ED-DBD reactor exhibited an increase, with the pulse amplitude attaining its maximum at the current peak. This was likely due to the formation of a new electric field caused by the electrons adhering to the stainless-steel rods.When SiO_2_ beads were introduced into the gap, the discharge intensity was further augmented as surface discharge occurs on the SiO_2_ bead surface (Mujahid and Hala, 2018). As shown in Figure 3C, the amplitude of the current pulse increased, and a secondary current pulse occurred before the main current pulse due to surface discharge. On the other hand, in the case of SiC blocks filled, the amplitude of the main current pulse was maintained, while the amplitude of the secondary current pulses increased, and their number was greater than that in the case of SiO_2_ beads, as depicted in Figure 3D. This was probably because the rougher surface of SiC could withstand a higher maximum current density than glass (Wang et al., 2015), resulting in more current pulses under the same discharge voltage.

**FIGURE 4:**
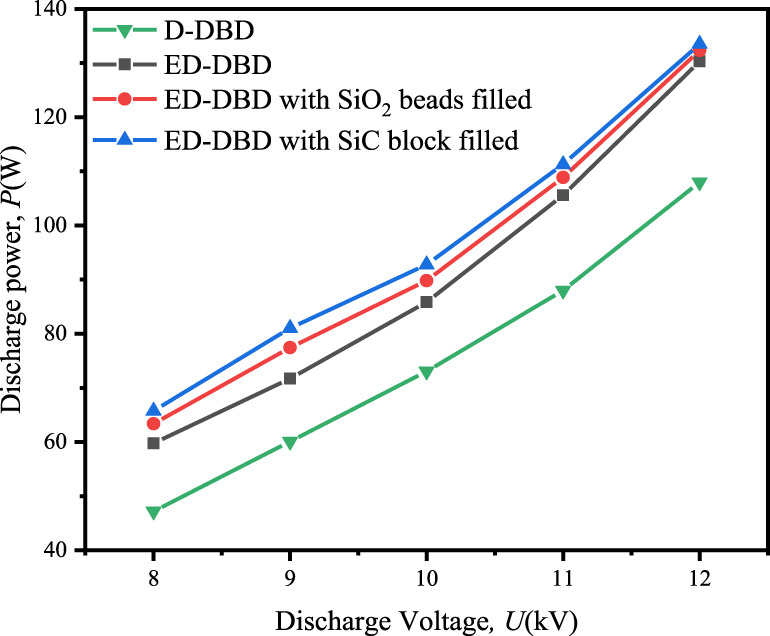
Effect of discharge voltage on the discharge power in D-DBD reactor, ED-DBD reactor, ED-DBD reactor (filled with SiO_2_ beads), and ED-DBD reactor (filled with SiC blocks).

The variation in discharge power with discharge voltage under different conditions was shown in Figure 4. For ED-DBD reactor, the discharge power could range from 59.8 W to 130.3 W for voltages ranging from 8 kV to 12.2 kV, while the range was 47.2–108.0 W for the D-DBD reactor. The partially narrowed gap caused by the second ground electrode in the ED-DBD reactor strengthened the local electric field and improved the discharge intensity, as shown in Figure 3. The discharge power was further enhanced by filling the reactor with insulating materials beads. For SiO_2_ beads and SiC blocks, the discharge powers ranged from 63.4 W to 132.3 W and 65.8 W to 133.5 W, respectively. The maximum discharge power increased by 1.5% and 2.5%, respectively, compared to that without packing. Based on the above analysis, the ED-DBD reactor had the advantage of higher discharge power and more discharge current pulses compared to the D-DBD reactor. Therefore, subsequent experiments only focused on ED-DBD reactor under different filling conditions.

**FIGURE 5:**
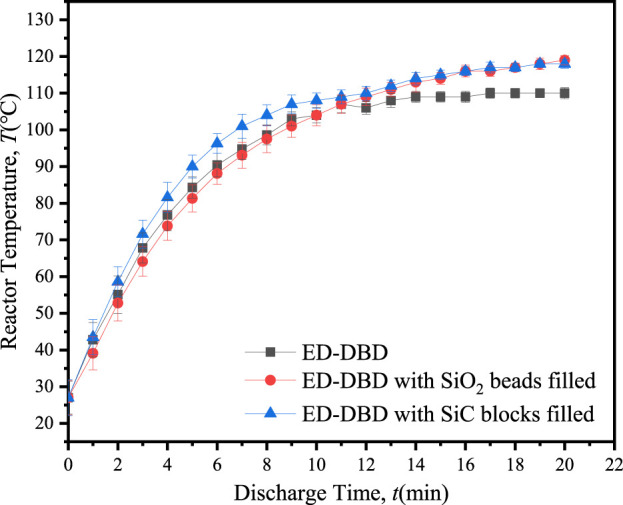
Variation of reactor temperature with discharge time in ED-DBD reactor, ED-DBD reactor (filled with SiO_2_ beads), and ED-DBD reactor (filled with SiC blocks).

The temperature of the reactor is a critical parameter for assessing the conversion of electrical energy during the discharge process. An elevated reactor temperature signified a greater proportion of energy being converted into heat, thereby diminishing energy efficiency (Duan et al., 2021). Figure 5 demonstrated the variation in reactor temperature with discharge time under different conditions. As time advanced, heat energy accumulated within the discharge gap, leading to a gradual increase in reactor temperature. After 20 min of discharge, the temperature stabilized, following the order: *T*_(SiC blocks in ED-DBD)_ = *T*_(SiO_2_ beads in ED-DBD)_ > *T*_(ED-DBD)_ under different conditions. In comparison to the case without packing material, the maintaining temperature of packing with SiO_2_ beads and SiC blocks increased by about 9.1%. When the reactor was filled with SiC or SiO_2_, the generated heat could be transferred to the packing medium, which had a higher specific heat capacity than air under normal temperature. Therefore, under the same discharge conditions, the temperature of the ED-DBD reactor containing SiO_2_ and SiC-filled packing was higher than that of the ED-DBD reactor without packing material.

### 3.2 Conversions of benzene and CO_2_

**FIGURE 6:**
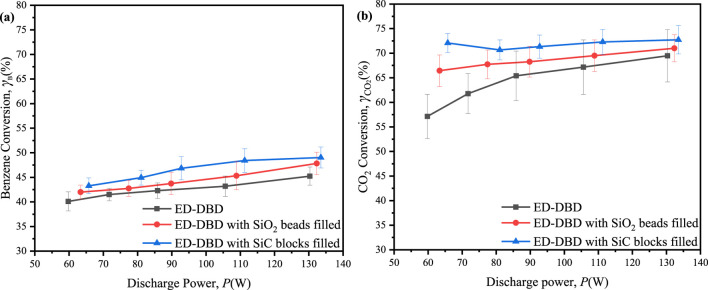
Effect of the discharge power on conversion rates of benzene and CO_2_ in ED-DBD reactor, ED-DBD reactor (SiO_2_ beads filled) and ED-DBD reactor (SiC blocks filled) under the initial concentration of benzene and CO_2_ are 300 ppm and 3,000 ppm, respectively. **(a)** conversion of benzene; **(b)** conversion of CO_2_.

The conversion of benzene and CO_2_ varied with discharge voltage given initial concentrations of 300 ppm for benzene and 3,000 ppm for CO_2_, as illustrated in Figure 6. An increase in discharge voltage resulted in benzene conversion efficiencies ranging from 40% to 50% (Figure 6A). The highest conversion efficiencies were observed at 49.0% with SiC block filling, 47.8% with SiO_2_ bead filling, and 45.2% without filling. The improved benzene conversion in the reactor containing packing materials could be attributed to the synergistic effects of surface discharge and prolonged residence time, which impeded the gas flow within the discharge region. The benzene degradation reached its maximum when the SiC blocks were utilized, due to the largest number of discharge current pulses. On the other hand, the maximum conversion of CO_2_ exhibited a notable increase, reaching 72.7% with SiC blocks, 71.0% with SiO_2_ beads, and 69.5% in the absence of any filing material (Figure 6B). The incorporation of packing materials within the reactor was advantageous for plasma generation, resulting in enhanced discharge intensity in the ED-DBD reactor and higher CO_2_ conversion rates. Interestingly, the CO_2_ conversion in the ED-DBD reactor filled with SiO_2_ beads and SiC blocks exhibited minimal dependence on the driving voltage. This was likely because benzene conversion was primarily influenced by reactive species generated from CO_2_ in the plasma, which were not directly associated with the applied voltages (Alliati et al., 2018).

**FIGURE 7:**
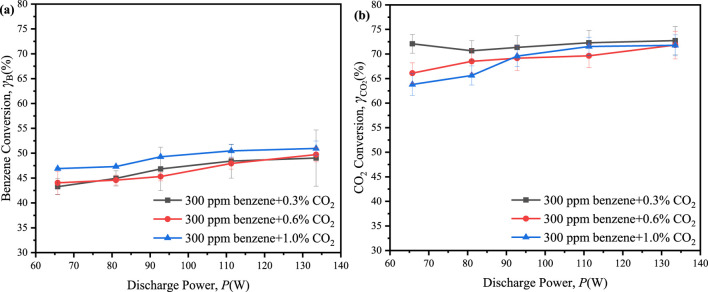
Effect of the discharge power on conversion of benzene and CO_2_ in ED-DBD reactor (SiC blocks filled) under the initial benzene concentration is 300 ppm and CO_2_ concentration are 0.3%, 0.6% and 1.0%, respectively. **(a)** conversion of benzene; **(b)** conversion of CO_2_.

To investigate the influence of CO_2_ concentration on benzene reforming, the reaction system was subjected to CO_2_ concentrations of 0.6% and 1.0%, alongside 300 ppm of benzene. The results indicated a slight increase in the maximum conversion of benzene, reaching 51.0% with 1.0% CO_2_ and 49.7% with 0.6% CO_2_, compared to 49.0% with 0.3% CO_2_, while the maximum conversion of CO_2_ presented a slight decline, decreasing from 72.7% at 0.3% CO_2_ to 71.8% at 0.6% CO_2_ and 71.7% at 1.0% CO_2_ (Figure 7). The presence of benzene facilitated interactions between reactive species derived from CO_2_ and benzene or its intermediates, resulting in the consumption of active species and enhancing the conversion of benzene and CO_2_. However, CO_2_ also competed with benzene for active particles (Xu et al., 2021; Zhu et al., 2020). When the concentration of CO_2_ reached a sufficiently high level, this competitive interaction was intensified, thereby diminishing the facilitative effect of CO_2_ on benzene degradation.

**FIGURE 8:**
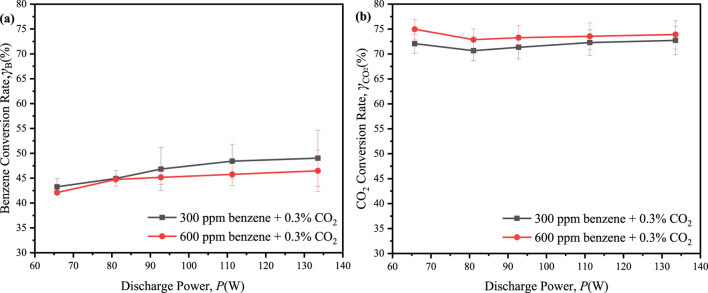
Effect of the discharge power on conversion of benzene and CO_2_ in the initial CO_2_ concentration is 0.3% and benzene concentration are 300 ppm, 600 ppm, respectively. **(a)** conversion of benzene; **(b)** conversion of CO_2_.

To further understand the impact of benzene concentration, we continuously introduced 600 ppm of benzene with 0.3% CO_2_ into the reaction system. The performances of benzene and CO_2_ conversion were depicted in Figure 8. The maximum conversion of benzene slightly decreased from 49.0% at 300 ppm benzene to 46.5% at 600 ppm benzene, while the maximum conversion of CO_2_ showed a modest increase from 72.7% at 300 ppm benzene to 75.0% at 600 ppm benzene upon the adding more benzene to the reaction system. It suggested that the increased presence of hydrogen atom donors from benzene facilitated the conversion of CO_2_, albeit at the expense of a reduced benzene conversion. Notably, CO_2_ conversion exceeded 60% across all ED-DBD reactors with SiC blocks, indicating potential suitability for industrial applications (Olivier et al., 2023).

### 3.3 Molar yields of CO and H_2_

**FIGURE 9:**
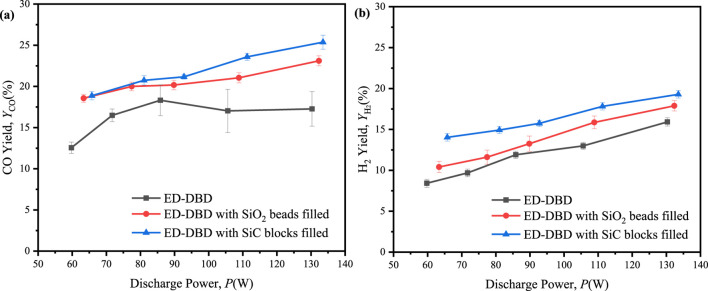
Effect of the discharge power on molar yields of syngas in ED-DBD reactor, ED-DBD reactor (SiO_2_ beads filled), ED-DBD reactor (SiC blocks filled) under the initial concentration of benzene is 300 ppm and the initial concentration of CO_2_ is 0.3%. **(a)** molar yields CO; **(b)** molar yields of H_2_.

Further investigation was conducted to assess the yield of gas-phase inorganic products in the reaction system characterized by an initial concentration of 300 ppm benzene and 3,000 ppm CO_2_, as shown in Figure 9. The analysis utilizing infrared (IR) spectroscopy and gas chromatography (GC) indicated that the primary products of benzene CO_2_ reforming included CO, H_2_, CH_4_ and H_2_O. The maximum CO yields obtaind in the ED-DBD reactor filled with SiC blocks recorded at 26.5%, representing 3% and 10% increase compared to the yields in the SiO_2_-filled ED-DBD reactor and blank ED-DBD reactor, respectively. Similarly, the maximum H_2_ yields in the ED-DBD reactor filled with SiC blocks were 19.2% and about 3% and 5% higher than the ED-DBD reactor with SiO_2_ beads filled and the ED-DBD reactor, respectively. When the discharge power gradually increased from 60.0 W to 130 W in the ED-DBD reactor with SiO_2_ beads filled, the CO yield increased slowly from 18.9% to 23.6% and the H_2_ yield slightly increased from 10.0% to 17.8%. These findings suggested that enhancing discharge power might not be the primary factor in improving the yields of H_2_ and CO, as evidenced by the nearly identical growth rates of CO and H_2_.

**FIGURE 10:**
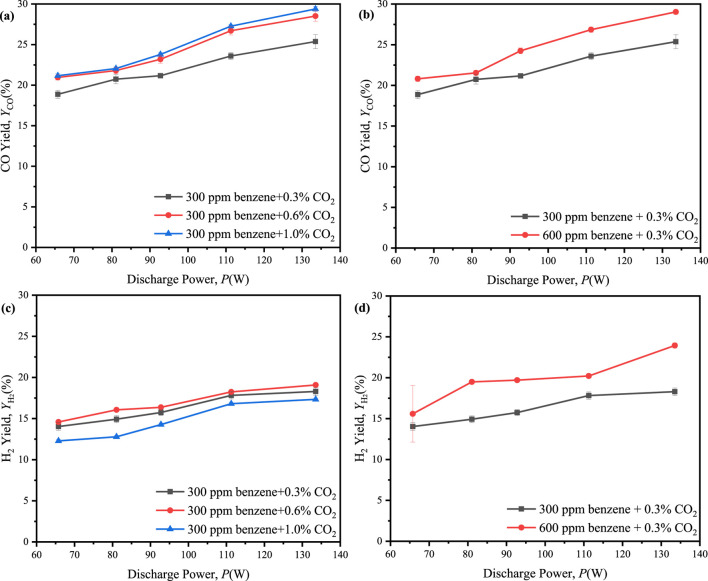
Effect of the discharge power on molar yields of CO and H_2_ in ED-DBD reactor (SiC blocks filled) under the different initial concentration. **(a, c)** the initial concentration of benzene is 300 ppm and the initial concentration of CO_2_ are 0.3%,0.6%,1.0%, respectively. **(b, d)** the initial concentration of CO_2_ is 0.3% and the initial concentration of benzene are 300 ppm and 600 ppm, respectively.

Furthermore, the introduction of 0.6% and 1.0% CO2, along with 300 ppm benzene, into the reaction system resulted in the maximum CO yield of 29.4% at 1.0% CO_2_, while a maximum H_2_ yield of 19.1% at 0.6% CO_2_, as shown in Figure 10. These results indicated that an increase in the initial concentration of CO_2_ could substantially enhance the molar yields of CO and H_2_. However, an excessive increase in the initial concentration of CO_2_ might lead to a reduction in the molar yield of H_2_. Additionally, the maximum molar ratio of H_2_/CO decreased from 0.135 to 0.045 as the initial concentration of CO_2_ increases from 0.3% to 1.0%, as shown in Figure 12A. It suggested that the initial concentration of CO_2_ was excessively high, resulting in a more rapid growth rate of CO compared to H_2_.

Figures 10B, D presented the yields of CO and H_2_ within a reaction system containing 600 ppm benzene and 0.3% CO_2_. In this system, the maximum yields of CO and H_2_ were observed at 29.0% and 24.0%, respectively, suggesting that an increase in the initial concentration of benzene could substantially enhance the molar yields of both CO and H_2_. Furthermore, as depicted in Figures 10B, D, the maximum molar ratio of H_2_/CO increased from 0.135 to 0.226 as the initial benzene concentration raise from 300 ppm to 600 ppm. It was indicated that the generation rate of H_2_ could surpass that of CO with the increased initial concentration of benzene.

**FIGURE 11:**
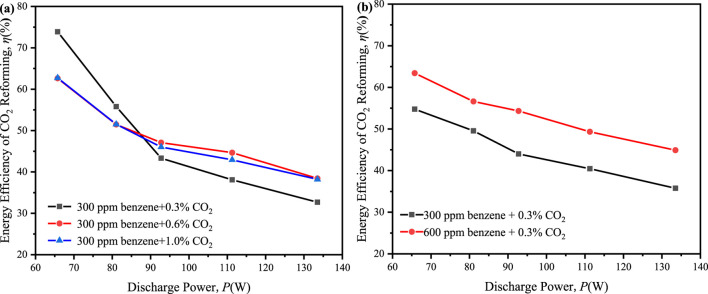
Effect of discharge power on the energy efficiency of CO_2_ reforming in ED-DBD reactor (SiC blocks filled). **(a)** in the initial concentration of benzene is 300 ppm and the initial concentration of CO_2_ are 0.3%, 0.6%, 1.0%, respectively; **(b)** in the initial concentration of CO_2_ is 0.3% and the initial concentration of benzene are 300 ppm and 600 ppm, respectively.

Finally, the energy efficiency of 32%–75% was achieved in all the ED-DBD reactor system which filled SiC blocks (Figure 11), exceeding the thresholds of 60% for future development of CO_2_ utilization (Olivier et al., 2023).

### 3.4 The role of SiC

**FIGURE 12:**
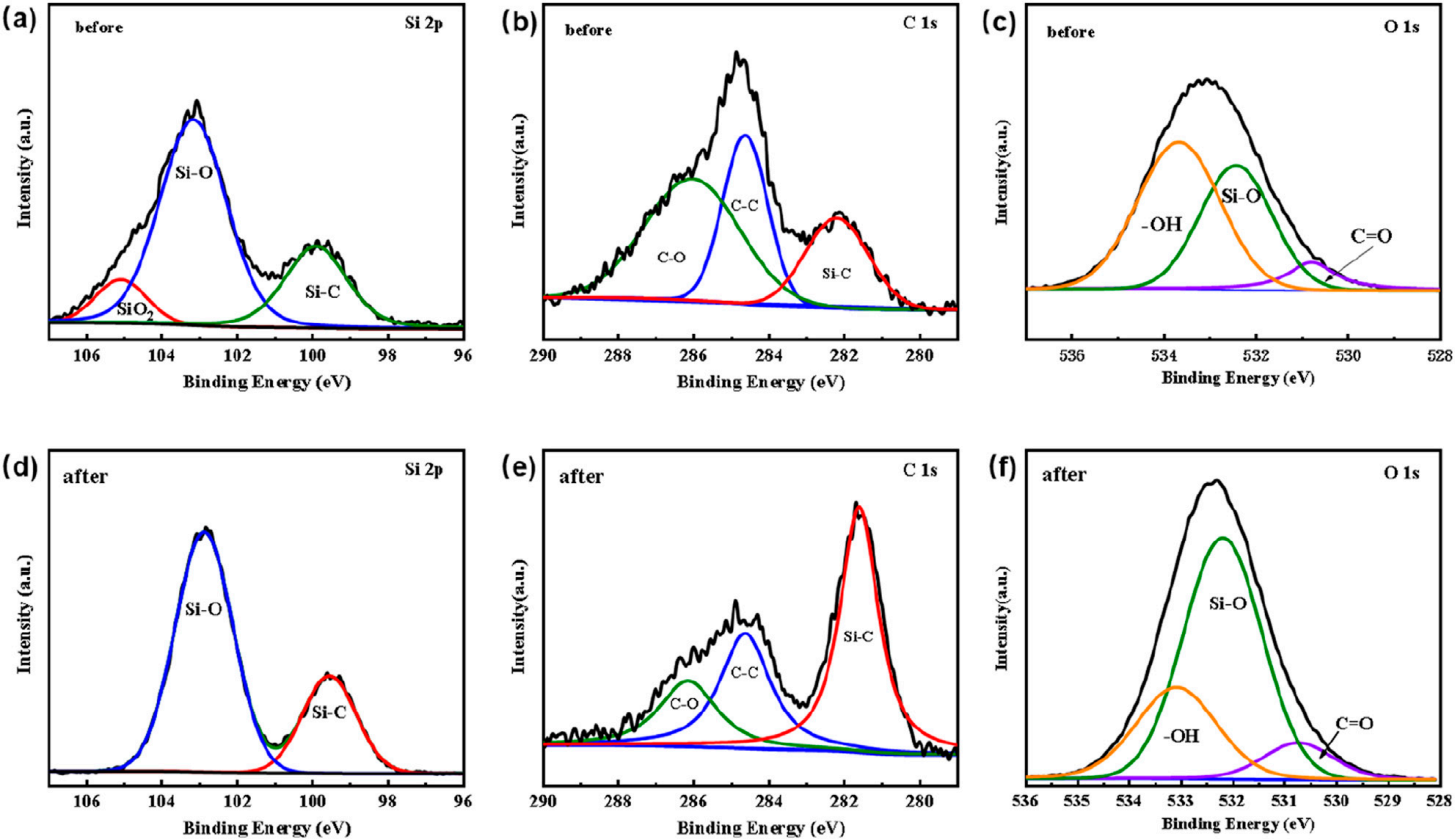
XPS spectrum of SiC blocks filled in ED-DBD reactor before and after reaction. **(a)** Si 2p, **(b)** C 1s and **(c)** O 1s XPS spectra of SiC blocks before use; **(d)** Si 2p, **(e)** C 1s and **(f)** O 1s of SiC blocks after use.

To verify the role of SiC, X-ray photoelectron spectroscopy (XPS) measurements were conducted to examinte the chemical states of Si and C in the SiC blocks, both prior to and following plasma exposure. Figures 12A, D showed the XPS spectra of Si 2p orbital before and after utilization, indicating the presence of Si-C bonds at approximately 99.5 eV. There was also a common Si-O peak at about 103.2 eV, indicating the presence of an oxide layer (SiO_x_) on the SiC surface due to oxidation (Charpentier et al., 2012; Tamayo et al., 2018). Previous studies have documented a significant peak at about 105 eV for pre-use SiC samples, attributed to processes. (Hollering et al., 1999; Önneby and Pantano, 1997). However, this peak at 105 eV disappears after utilization, as the plasma bombardment facilitates the exposure of a new SiC layer. The C1s resulted in Figures 12B, E further confirm this, showing a significant increase in the proportion of -C bonds with plasma reaction. Combining with findings in Figure 6, it could be inferred that a small amount of C elements from the SiC blocks in the plasma region were involved in the reaction process on the SiC surface. Meanwhile, the O 1s XPS spectra depicted in Figures 12C, F indicated a substantial increase in the proportion of Si-O bonds following plasma treatment. The above observation suggested that Si within the packing materials played a crucial role in the CO_2_ reforming of benzene. The detailed mechanisms were still under investigation.

### 3.5 Optical emission spectroscopy

**FIGURE 13:**
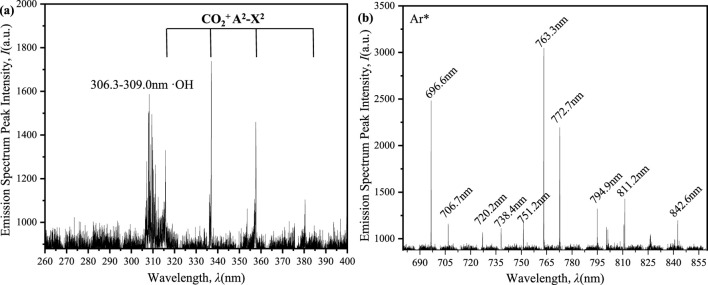
Emission spectra of ED-DBD reactor (SiC blocks filled) plasma at an applied voltage of 12 kV and a frequency of 8 kHz. **(a)** in the range of 200–400nm; **(b)** in the range of 650–900 nm.

To gain the insights into the discharge process in the SiC-packed ED-DBD reactor, optical emission spectroscopy was performed at a voltage amplitude of 12 kV and atmospheric pressure. As depicted in Figure 13, the emission spectral lines were mainly observed in two regions: 200–400 nm and 700–850 nm. The OH (A^2^∑^+^→X^2^∏) emission band (306.3–309.0 nm) was clearly identified, along with weaker CO_2_^+^(A^2^→X^2^) emission bands at 325 nm, 337 nm, 355 nm, and 370 nm. Additionally, prominent Ar (2P→1S) emission lines were observed at wavelengths of 696.6 nm, 706.7 nm, 720.2nm, 738.4 nm, 751.2 nm, 763.3 nm, 772.7 nm, 794.9 nm, 811.2 nm and 842.6 nm.

**FIGURE 14:**
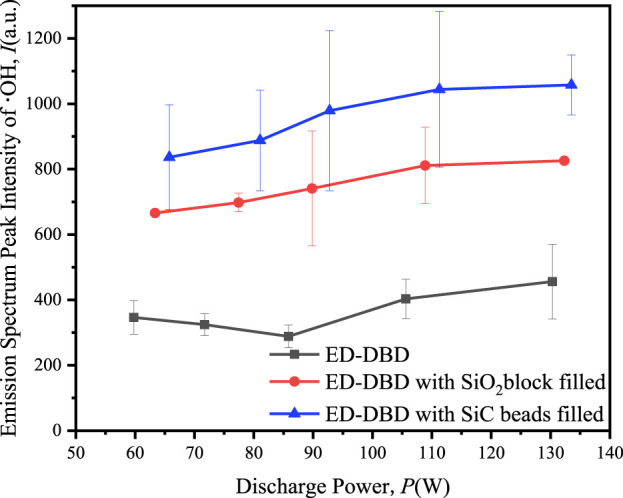
Effect of discharge power on emission spectrum peak intensity in ED-DBD reactor, ED-DBD reactor (SiO_2_ beads filled), and ED-DBD reactor (SiC blocks filled).

By utilizing emission spectroscopy, the relative intensities of the emission lines emitted by the active OH species were evaluated with applied voltages (Figure 14). The results indicated that the intensities of OH emission lines in the SiC and SiO_2_ packed ED-DBD reactor were higher than those in the blank ED-DBD reactor, with a slight increase in intensity as the applied power increased from 60 W to 120 W. Furthermore, the OH emission intensity in the SiC-packed ED-DBD reactor was higher than in the SiO_2_ beads filled reactor. This might serve as indirect evidence suggesting that the SiC-packed ED-DBD reactor performed better in the CO_2_ reforming of benzene compared to the SiO_2_ beads.

## 4 Conclusion

Dielectric barrier discharge presented extensive potential for application in the conversion of biomass tar. To explore the CO_2_ reforming of benzene, serving as a tar analog in biomass gasification, ED-DBD reactors incorporating SiO_2_ beads and SiC blocks were developed. Firstly, the addition of SiC filling significantly improved the reaction efficiency of ED-DBD reactor. Under the same discharge parameters, the SiC-filled reactor exhibited lower discharge power compared to the unfilled case, while achieving a discharge current pulse six times higher and a CO_2_ conversion rate more than 1.3 times higher. XPS analysis revealed that a small quantity of C elements from the SiC blocks in the plasma region participated in the reaction process, which likely facilitated the degradation reaction. Secondly, the ED-DBD reactor filled with SiC blocks demonstrated the best performance in CO_2_ reforming of benzene. It achieved a benzene conversion of 51.0%, CO_2_ conversion of 75.0%, energy efficiency of CO_2_ conversion of 73.9%. These results at least met the minimum requirements for conversion rate and energy efficiency of CO_2_ oxidation biomass gasification tar to syngas industrialization. Moreover, increasing initial concentration of CO_2_ in the benzene system promoted the benzene conversion, while adding benzene to the CO_2_ system facilitated the conversion of CO_2_. Additionally, the SiC-packed ED-DBD reactor was found to produce active OH particles during the discharge process, as detected using emission spectroscopy. This study not only presented an effective method for converting tar analogues into syngas under mild conditions but also offered an alternative approach for CO_2_ utilization within a carbon-neutral strategy.

## Data Availability

The raw data supporting the conclusions of this article will be made available by the authors, without undue reservation.
